# Advancing Health Equity in Alzheimer's Economic Evaluations ‐ Leveraging DGRACE Model for Improved Outcomes

**DOI:** 10.1002/alz70860_107775

**Published:** 2025-12-23

**Authors:** Emmanuel F Drabo, Michael Michael DiStefano2, Jacqualine Woo, Shanshan Lin, Ravi Gupta, Renee F Wilson, Halima Amjad, Emily Mao, Jodi B Segal

**Affiliations:** ^1^ Johns Hopkins, Baltimore, MD, USA; ^2^ University of Colorado Anschutz Skaggs School of Pharmacy and Pharmaceutical Sciences, Aurora, CO, USA; ^3^ Johns Hopkins University, Baltimore, MD, USA; ^4^ slin95@jhu.edu, Baltimore, MD, USA; ^5^ Johns Hopkins University School of Medicine, Baltimore, MD, USA; ^6^ Center on Aging and Health, Baltimore, MD, USA

## Abstract

**Background:**

Emerging treatments for Alzheimer's disease and related dementias (ADRD) provide moderate health benefits but are costly and pose risks of severe adverse events. Traditional cost‐effectiveness analyses (CEAs) often deem these therapies not cost‐effective at conventional willingness‐to‐pay (WTP) thresholds. However, standard CEA overlooks factors such as disease severity, pre‐existing disabilities, and health equity. The generalized risk‐adjusted cost‐effectiveness (GRACE) model addresses some limitations by incorporating diminishing health returns and variable WTP thresholds but does not directly account for social inequities. Here, we propose the distributional GRACE (DGRACE) model, which extends GRACE by incorporating equity considerations.

**Methods:**

We developed DGRACE by adjusting GRACE's quality‐adjusted life years (QALYs) and WTP thresholds to capture the social distribution of intervention effects. We applied the model to two diseases with differing burden distributions in older U.S. adults (≥65 years): ADRD, with a highly unequal disease burden, and idiopathic pulmonary fibrosis (IPF), with a relatively uniform burden. Varieties of utility functions (e.g., isoelastic, expo‐power), inequality indices (e.g., Atkinson, Gini), and inequality aversion parameters were explored to assess their effects on treatment prioritization. Results were compared with GRACE, standard CEA, and distributional CEA.

**Results:**

DGRACE shifted treatment prioritization toward diseases with greater population burden inequalities, such as ADRD, compared to IPF. In simulations without inequality aversion or with evenly distributed treatment effects, DGRACE aligned with GRACE, yielding similar QALYs and WTP thresholds. Higher inequality aversion increasingly prioritized treatments for conditions with greater inequities, highlighting ADRD as a key priority.

**Conclusion:**

DGRACE offers a novel framework for integrating health equity into economic evaluations of emerging ADRD treatments. It highlights the potential for equity‐informed prioritization in healthcare decision‐making. Further research is needed to refine social inequality metrics, define optimal inequality aversion parameters, and identify suitable utility function specifications to advance health equity.

